# Concurrent *Nocardia*, *Cryptococcus* and *Mycobacterium* Infections Unmask Anti–GM‐CSF Antibody–Associated Immunodeficiency and Pulmonary Alveolar Proteinosis: A Case Report

**DOI:** 10.1155/crdi/5939262

**Published:** 2026-06-23

**Authors:** Vidthiya Menon

**Affiliations:** ^1^ Nepean Clinical School, The University of Sydney, Sydney, New South Wales, Australia, sydney.edu.au; ^2^ Department of Infectious Diseases and Microbiology, Nepean Hospital, Penrith, New South Wales, Australia, nbmlhd.health.nsw.gov.au

**Keywords:** anti–GM-CSF antibodies, case report, *Cryptococcus*, mycobacteria, *Nocardia*

## Abstract

Anti‐granulocyte macrophage colony stimulating factor (anti‐GM‐CSF) antibodies, classically associated with pulmonary alveolar proteinosis (PAP), are increasingly recognised as a cause of adult‐onset immunodeficiency predisposing to opportunistic infections. Coinfections with multiple opportunistic pathogens in this context are uncommon. We describe a rare case of disseminated *Nocardia paucivorans*, pulmonary *Cryptococcus gattii,* pulmonary *Mycobacterium chelonae*, and subsequent PAP in a patient with high‐level anti–GM‐CSF antibodies. A 64‐year‐old man presented with subacute bilateral shoulder pain and was diagnosed with acromioclavicular septic arthritis. *N. paucivorans* was isolated, and subsequent evaluation demonstrated disseminated infection with numerous brain abscesses, left eye endophthalmitis and pulmonary involvement. Interval computed tomography of the chest revealed new right lower lobe consolidation, a biopsy of which identified *C. gattii* and *M. chelonae*. Immunological testing confirmed high‐level anti–GM‐CSF antibodies. The patient received prolonged combination antimicrobial therapy, including meropenem, ceftriaxone, linezolid, trimethoprim‐sulfamethoxazole, moxifloxacin, fluconazole, tigecycline and clofazimine, with clinical and radiological improvement of infectious lesions. Despite microbiological clearance, progressive bilateral ground‐glass opacities developed on serial chest imaging consistent with PAP, with no pathogens identified on bronchoscopic sampling. Given minimal respiratory symptoms, PAP‐directed therapy was deferred. The patient remains clinically stable on trimethoprim‐sulfamethoxazole prophylaxis with ongoing clinical and radiological surveillance. This case illustrates the expanding clinical spectrum of anti–GM‐CSF antibody‐associated disease and underscores the importance of considering this diagnosis in patients presenting with opportunistic infections, in particular, disseminated nocardiosis or *C*. *gattii* infection. It also highlights the need for vigilance in evaluating for coinfections, recognition of PAP as a noninfectious codiagnosis, and the importance of long‐term follow‐up in affected patients.

## 1. Introduction

Neutralising autoantibodies against cytokines are increasingly recognised as causes of immunodeficiencies. Well‐described associations include anti–interferon‐γ antibodies with disseminated nontuberculous mycobacterial and invasive nontyphoidal *Salmonella* infections [1], anti–IL‐6 antibodies with severe staphylococcal and pneumococcal infections [2], and anti–IL‐17 antibodies with chronic mucocutaneous candidiasis [3]. The mechanisms underlying the development of anticytokine antibodies remain unclear, and several have been associated with autoimmune disease [4].

GM‐CSF is a cytokine produced by various cells, including alveolar epithelial cells, monocytes and macrophages, which plays a critical role in the differentiation and function of macrophages and granulocytes, particularly alveolar macrophages. Neutralisation of GM‐CSF impairs macrophage chemotaxis, phagocytosis, intracellular killing, and surfactant clearance, predisposing affected individuals to infections with inhaled pathogens, including *Nocardia* and *Cryptococcus*, as well as to the development of pulmonary alveolar proteinosis (PAP) [4–6]. Although individual infections associated with anti–GM‐CSF antibodies are increasingly recognised, concurrent infections with multiple opportunistic pathogens are rare.

An earlier report described a limited aspect of this patient’s clinical course, focusing on ocular and cerebral *Nocardia paucivorans* infection in a patient initially considered immunocompetent [7]. The current report expands upon these findings by describing concurrent *Cryptococcus gattii* and *Mycobacterium chelonae* infections, identification of underlying anti–GM‐CSF antibody–associated immunodeficiency, and the later development of PAP, providing a comprehensive longitudinal perspective of a rare constellation of diagnoses.

## 2. Case Presentation

A 64‐year‐old previously healthy man presented in July 2021 with several weeks of bilateral shoulder pain and fever. He was diagnosed with bilateral acromioclavicular joint septic arthritis requiring surgical washouts. Postoperatively, he developed acute confusion. Computed tomography (CT) and magnetic resonance imaging (MRI) of the brain demonstrated multiple subcentimetre intracranial abscesses (Figure 1). Gram stain of operative specimens from both shoulders demonstrated beaded Gram‐positive rods, and cultures subsequently identified *Nocardia paucivorans*. The patient also reported floaters in the vision of his left eye and was diagnosed with endogenous endophthalmitis, presumed secondary to nocardial dissemination. Vitreous fluid cultures were negative, having been obtained after commencement of antimicrobial therapy. CT of the chest demonstrated hilar lymphadenopathy and a right perihilar density, also attributed to nocardial infection. He was treated with prolonged combination antimicrobial therapy, ultimately guided by susceptibility testing, including various combinations of meropenem, trimethoprim‐sulfamethoxazole, ceftriaxone, moxifloxacin and linezolid, with clinical and radiological improvement. Initial investigations did not identify an underlying immunodeficiency including HIV testing, lymphocyte subsets and immunoglobulin levels. The initial ocular and cerebral nocardial infection previously reported [7] is summarised here to provide clinical context; all subsequent infections, immunological findings, and follow‐up described in this report are novel.

**FIGURE 1:**
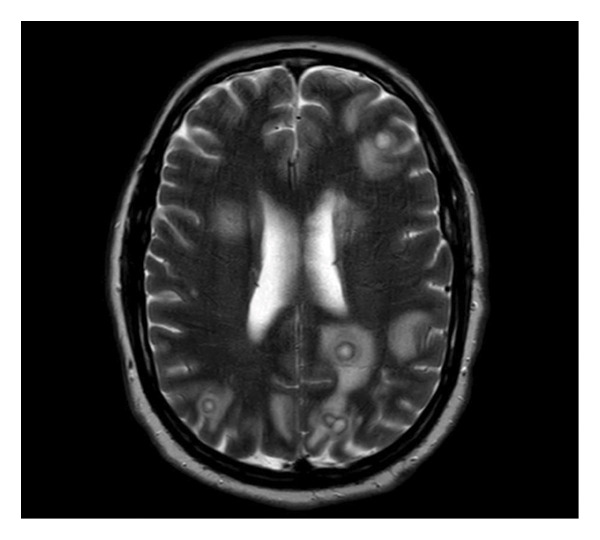
Initial magnetic resonance imaging of the brain showing multiple brain abscesses with surrounding vasogenic oedema.

Nine months into treatment, interval chest imaging demonstrated a new region of consolidation in the right lower lobe (Figure 2). Serum cryptococcal antigen testing was positive, with titres rising to 1:2560. CT‐guided lung biopsy demonstrated interstitial and granulomatous inflammation with organisms morphologically consistent with *Cryptococcus*. Cryptococcal PCR was positive for *Cryptococcus gattii*; however, fungal cultures were negative. Cultures from the lung biopsy grew *Mycobacterium chelonae*, although mycobacterial PCR was negative. *Mycobacterium chelonae* was felt to be a contributing pathogen, given the limitations of PCR testing, and Infectious Diseases Society of America diagnostic criteria for nontuberculous mycobacterial pulmonary disease were met, given clinical and radiological findings, together with granulomatous inflammation and a positive culture [8]. Nocardial cultures were negative. Lumbar puncture revealed mildly elevated protein (0.67 g/L) and 2 mononuclear cells, with negative cryptococcal antigen, PCR and fungal cultures. Further evaluation for immunodeficiency revealed high levels of anti–GM‐CSF antibodies, detected by enzyme‐linked immunosorbent assay (ELISA), and confirmed on repeat testing 3 months later. The patient was treated with further prolonged combination antimicrobial therapy, including various combinations of fluconazole, linezolid, tigecycline, clarithromycin, trimethoprim‐sulfamethoxazole and clofazimine. Treatment was complicated by linezolid‐induced peripheral neuropathy, clofazimine‐associated skin hyperpigmentation and QTc prolongation requiring close monitoring.

**FIGURE 2:**
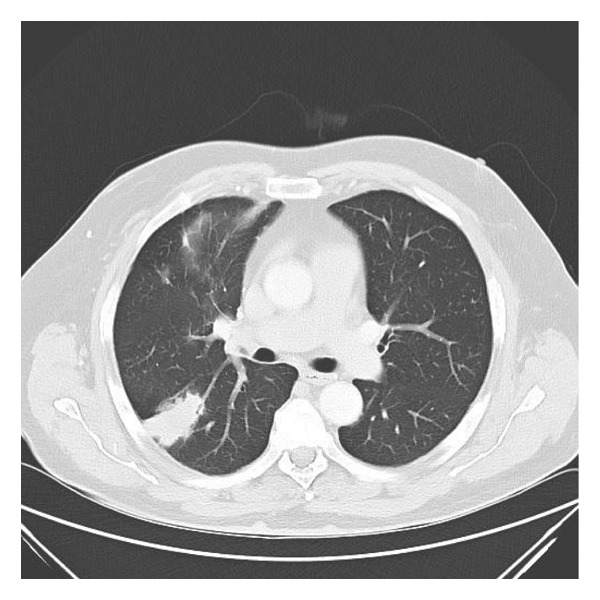
New right lower lobe consolidation on progress computed tomography imaging 9 months into treatment for disseminated nocardiosis. Some ground‐glass change is seen in the anterior right upper lobe.

After 18 months of therapy for *N. paucivorans*, 20 months for *C. gattii*, and 18 months for *M. chelonae*, the patient demonstrated marked clinical and radiological improvement. Residual sequelae included persistent left‐sided visual impairment, peripheral neuropathy and a mild chronic cough. He remains clinically stable on prophylactic trimethoprim‐sulfamethoxazole alone, 18 months after completion of active treatment.

Eighteen months after initial presentation, however, new right upper‐lobe ground‐glass opacities were identified on chest CT imaging. Bronchoscopy with bronchoalveolar lavage was negative for nocardial, fungal and mycobacterial pathogens, and cytology showed no evidence of malignancy. Periodic acid–Schiff staining was not performed at this time. Over the subsequent 3 years, progressive bilateral ground‐glass opacities developed, with some areas showing ‘crazy paving’ (Figure 3). In the context of anti–GM‐CSF antibodies and radiological progression, PAP was considered the most likely diagnosis despite lack of further confirmatory testing. Given minimal symptoms, further evaluation and specific therapy have not been initiated, and the patient remains under ongoing clinical and radiological surveillance.

**FIGURE 3:**
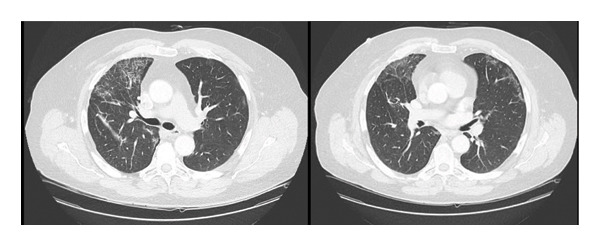
Diffuse ground‐glass changes involving all lobes with predominantly upper and anterior distribution, some areas showing crazy paving, demonstrated on progress computed tomography imaging of the chest post completion of active treatment of infection.

## 3. Discussion

Anti–GM‐CSF antibody‐associated disease is a complex and evolving immunodeficiency syndrome, increasingly recognised for its association with opportunistic infections such as nocardiosis and cryptococcosis, with possible mechanisms increasingly elucidated. Neutralising antibodies disrupt GM‐CSF‐dependent immune pathways, including STAT5 phosphorylation in response to *Nocardia*, and impair macrophage survival and intracellular control of *Mycobacterium tuberculosis* [9, 10]. Furthermore, defective neutrophil activation and reduced reactive oxygen species production contribute to susceptibility to invasive fungal infections such as aspergillosis [11]. Collectively, these findings underscore the vital role of GM‐CSF in host defence and highlight some of the immunological consequences of its neutralisation.

Anti–GM‐CSF antibodies can be detected using ELISA, although testing is not widely available [4]. Low levels may be present in healthy individuals, whereas higher levels have been associated with PAP, disseminated and central nervous system (CNS) infections as well as increased infection‐related hospitalisations [6, 12, 13]. Antibodies have also been found in cerebrospinal fluid, although the relationship with CNS infection remains unclear [6]. Importantly, antibody levels do not consistently correlate with PAP disease activity or therapeutic response to PAP directed treatment, though lower baselines or gradual declines are often noted in patients with spontaneously improving PAP [14].

Among patients with anti–GM‐CSF antibodies, *Nocardia* and *Cryptococcus* are the most frequently reported infections, typically as single pathogens [15–18]. Reported coinfections are uncommon (see Table 1). Nocardiosis often presents with CNS, pulmonary or disseminated involvement; a French case series reported CNS involvement in 70% of patients, pulmonary involvement in 60%, and disseminated involvement in 50% [15]. Cryptococcal infections in this population are more commonly caused by *Cryptococcus gattii* than *C. neoformans*, frequently involve the CNS and may present atypically with complications such as vasculitis. The presence of anti–GM‐CSF antibodies has been associated with lower survival in these patients [19–23].

**TABLE 1:** Reported cases of multiple opportunistic infections in patients with anti–GM‐CSF antibodies.

Reference	Age, sex	Infections	PAP reported	Outcome reported
Brillant‐marquis et al, 2025 [17]	56F	Nocardia, Actinomyces, *M. avium*	No	Recovered Trimethoprim‐sulfamethoxazole prophylaxis
Rosen et al., 2015 [9]	73M	Nocardia (cutaneous, pulmonary, CNS), *Aspergillus fumigatus* (pulmonary)	No	On‐going treatment with voriconazole, imipenem‐cilastatin, amikacin, subcutaneous GM‐CSF
Rosen et al., 2015 [9]	52F	*Nocardia asteroides* (pulmonary, CNS), disseminated cryptococcosis	No	Recovered No antimicrobials
Rosen et al., 2015 [9]	54F	*Nocardia beijingensis* (pulmonary, CNS), MAC, *Aspergillus* (pulmonary)	No	Unknown
Mabo et al., 2023 [13]	37M	Nocardia (pulmonary), *M. chimaera*	Yes	Unknown
Lo et al., 2024 [18]	64F	*N. beijingensis* (CNS), *M. abscessus*	No	Unknown
Lee et al., 2022 [16]	50M	*Flavobacterium*, *Nocardia* (CNS)	Yes	Recovered Trimethoprim‐sulfamethoxazole prophylaxis
Lee et al., 2022 [16]	48M	*C. neoformans* (pulmonary, CNS), *M. tuberculosis* (pulmonary)	No	Recovered

Impaired GM‐CSF‐dependent clearance of surfactant by dysfunctional alveolar macrophages can lead to PAP, characterised by accumulation of dense proteinaceous infiltrates causing ground‐glass changes and ‘crazy paving’ on CT, as was seen in this case. Most cases of PAP are adult‐onset and autoimmune, due to high levels of neutralising anti–GM‐CSF antibodies. Clinically PAP may present with progressive dyspnoea and secondary infections [24], and prognosis is variable. In a review of 104 cases, 28% of patients experienced spontaneous improvement, 67% required specific treatment, and the 5‐year survival rate was 95% [13].

Patients with anti–GM‐CSF antibodies may develop PAP, opportunistic infections, or both, with variable temporal relationships, complicating assessment of risk. Reported incidence of opportunistic infection in PAP ranges from 4% to 22% [4, 13]. In a review of 75 cases, PAP was the initial diagnosis in 33%, concurrent in 27% and subsequently diagnosed in 40% [25]. In the present case, PAP developed radiologically 18 months after the initial infectious presentation, underscoring the need for long‐term surveillance even after apparent resolution of infection.

Treatment options for PAP include whole lung lavage, adjunctive recombinant GM‐CSF and rituximab in refractory disease [13, 26]. PAP treatment likely contributes to prevention and clearance of pathogens; however, the extent to which it does is uncertain. Conversely, treatment of infection may impact PAP, with Wu et al. reporting a case of pulmonary nocardiosis together with PAP causing diffuse bilateral lung infiltration, which responded to antimicrobials alone [27]. Evidence to specifically guide management of infection risk in patients with anti–GM‐CSF antibodies is limited. Although recombinant GM‐CSF is a promising treatment for PAP, by restoring alveolar macrophage function, its utility in the treatment of infection is unclear, and evidence is limited to in‐vitro studies and case reports. GM‐CSF therapy has been shown to enhance cryptococcal clearance and has also been used successfully in disseminated nocardiosis [28]. Anti–CD‐20 monoclonal antibody rituximab can reduce the production of autoantibodies and has been shown to be effective in case reports of cytokine antibody‐associated nontuberculous mycobacterial infection [29]. Rituximab may represent a therapeutic option as an adjunct to treat anti–GM‐CSF antibody–associated infection in refractory cases. No formal guidelines exist regarding primary or secondary antimicrobial prophylaxis, although trimethoprim‐sulfamethoxazole should be considered, particularly given its activity against *Nocardia*. Longitudinal monitoring of antibody titres may inform risk stratification and therefore the frequency required for clinical, radiological and microbiological evaluation for infection, but further studies are needed to define its clinical utility.

This case highlights the importance of testing for anti–GM‐CSF antibodies in previously immunocompetent patients presenting with disseminated nocardiosis or *C. gattii* infection. Clinicians should actively consider coinfection and pursue sampling from multiple anatomical sites when indicated, with multiple modes of testing utilised given the varying specificity and sensitivity of culture, molecular testing and histopathology. Clinicians should also ensure long‐term multidisciplinary follow‐up involving infectious diseases, respiratory medicine and immunology given the on‐going risks of opportunistic infection and PAP in this patient population.

## Author Contributions

Vidthiya Menon oversaw and treated the case and authored the article.

Drafting and finalisation of work for publication, accountability for all aspects of work.

## Funding

The research did not receive any funding or financial support.

Open access publishing was facilitated by The University of Sydney, as part of the Wiley‐The University of Sydney agreement via the Council of Australasian University Librarians.

## Disclosure

An earlier, limited component of this case has been previously published [7]. The current manuscript reports new clinical findings, diagnoses, and longitudinal follow‐up not included in the prior publication.

## Ethics Statement

Ethical approval was not required for this case report. Written informed consent was obtained from the patient for publication of the case. All efforts have been made to ensure anonymity and confidentiality of the patient.

## Consent

Informed consent was obtained from the patient for publication.

## Conflicts of Interest

The author declares no conflicts of interest.

## Data Availability

Data sharing is not applicable to this article as no datasets were generated or analysed during the study.

## References

[1] Puel A. , Bastard P. , Bustamante J. , and Casanova J.-L. , Human Autoantibodies Underlying Infectious Diseases, Journal of Experimental Medicine. (2022) 219, no. 4, 10.1084/jem.20211387. PMC8952682 35319722

[2] Puel A. , Picard C. , Lorrot M. et al., Recurrent Staphylococcal Cellulitis and Subcutaneous Abscesses in a Child with Autoantibodies Against IL-6, Journal of Immunology. (2008) 180, no. 1, 647–654, 10.4049/jimmunol.180.1.647. 18097067

[3] Puel A. , Human Inborn Errors of Immunity Underlying Superficial or Invasive Candidiasis, Human Genetics. (2020) 139, no. 6–7, 1011–1022, 10.1007/s00439-020-02141-7. 32124012 PMC7275885

[4] Ataya A. , Knight V. , Carey B. C. , Lee E. , Tarling E. J. , and Wang T. , The Role of GM-CSF Autoantibodies in Infection and Autoimmune Pulmonary Alveolar Proteinosis: A Concise Review, Frontiers in Immunology. (2021) 12, 10.3389/fimmu.2021.752856. PMC8647160 34880857

[5] Zhan Y. , Xu Y. , and Lew A. M. , The Regulation of the Development and Function of Dendritic Cell Subsets by GM-CSF: More than a Hematopoietic Growth Factor, Molecular Immunology. (2012) 52, no. 1, 30–37, 10.1016/j.molimm.2012.04.009. 22580403

[6] Rosen L. B. , Freeman A. F. , Yang L. M. et al., Anti-GM-CSF Autoantibodies in Patients With Cryptococcal Meningitis, Journal of Immunology. (2013) 190, no. 8, 3959–3966, 10.4049/jimmunol.1202526. PMC3675663 23509356

[7] Thakkar H. , Raj P. R. , Howard-Jones A. R. , Adler P. , and Menon V. , *Nocardia paucivorans* Endophthalmitis and Cerebral Abscesses in an Immunocompetent Patient: a Case Report, Infectious Diseases in Clinical Practice. (2023) 31, no. 6, 1–5, 10.1097/ipc.0000000000001313.

[8] Daley C. L. , Iaccarino J. M. , Lange C. et al., Treatment of Nontuberculous Mycobacterial Pulmonary Disease: An Official ATS/ERS/ESCMID/IDSA Clinical Practice Guideline, Clinical Infectious Diseases. (2020) 71, no. 4, 905–913, 10.1093/cid/ciaa241. 32797222 PMC7768745

[9] Rosen L. B. , Rocha Pereira N. , Figueiredo C. et al., Nocardia-Induced Granulocyte Macrophage Colony-Stimulating Factor is Neutralized by Autoantibodies in Disseminated/Extrapulmonary Nocardiosis, Clinical Infectious Diseases. (2015) 60, no. 7, 1017–1025, 10.1093/cid/ciu968. 25472947 PMC4366584

[10] Mishra A. , Singh V. K. , Jagannath C. et al., Human Macrophages Exhibit GM-CSF-Dependent Restriction of *Mycobacterium tuberculosis* Infection via Regulating Their Self-Survival, Differentiation and Metabolism, Frontiers in Immunology. (2022) 13, 10.3389/fimmu.2022.859116. PMC9134823 35634283

[11] Wishart A. L. , Pechacek J. , Rosen L. B. et al., Neutralizing GM-CSF Autoantibodies Impair Neutrophil Antifungal Effector Function in a Patient With Aspergillosis, Journal of Infection. (2025) 91, no. 3, 10.1016/j.jinf.2025.106588. PMC12404695 40812454

[12] Salvator H. , Cheng A. , Rosen L. B. et al., Neutralizing GM-CSF Autoantibodies in Pulmonary Alveolar Proteinosis, Cryptococcal Meningitis and Severe Nocardiosis, Respiratory Research. (2022) 23, no. 1, 10.1186/s12931-022-02103-9. PMC9552154 36221098

[13] Mabo A. , Borie R. , Wemeau-Stervinou L. et al., Infections in Autoimmune Pulmonary Alveolar Proteinosis: A Large Retrospective Cohort, Thorax. (2023) 79, no. 1, 68–74, 10.1136/thorax-2023-220040. 37758458

[14] Nishimura M. , Yamaguchi E. , Takahashi A. et al., Clinical Significance of Serum Anti-GM-CSF Autoantibody Levels in Autoimmune Pulmonary Alveolar Proteinosis, Biomarkers in Medicine. (2018) 12, no. 2, 151–159, 10.2217/bmm-2017-0362. 29202602

[15] Kerdiles T. , Lejeune S. , Portais A. et al., Nocardia Infection in Patients With Anti-Granulocyte-Macrophage Colony-Stimulating Factor Autoantibodies: A Prospective Multicenter French Study, Open Forum Infectious Diseases. (2024) 11, no. 6, 10.1093/ofid/ofae269. PMC11194753 38915339

[16] Lee E. , Miller C. , Ataya A. , and Wang T. , Opportunistic Infection Associated With Elevated GM-CSF Autoantibodies: A Case Series and Review of the Literature, Open Forum Infectious Diseases. (2022) 9, no. 5, 10.1093/ofid/ofac146. PMC9070348 35531378

[17] Brillant-Marquis F. , Simard L. , and Marois L. , Nocardia Infections in 3 Patients With Anti-GM-CSF Autoantibodies, Journal of Allergy and Clinical Immunology. (2025) 155, no. 2, 10.1016/j.jaci.2024.12.094.

[18] Lo Y.-F. , Wang S.-Y. , Wu Y.-H. et al., The Pathogenic Role of Anti-Granulocyte-Macrophage Colony-Stimulating Factor Autoantibodies in Nocardiosis With Central Nervous System Involvement, Journal of Clinical Immunology. (2024) 44, no. 8, 10.1007/s10875-024-01775-w. 39133333

[19] Saijo T. , Chen J. , Chen S. C.-A. et al., Anti-Granulocyte-Macrophage Colony-Stimulating Factor Autoantibodies Are a Risk Factor for Central Nervous System Infection by Cryptococcus gattii in Otherwise Immunocompetent Patients, mBio. (2014) 5, no. 2, e00912–e00914, 10.1128/mbio.00912-14. 24643864 PMC3967522

[20] Jiang Y.-K. , Zhou L.-H. , Cheng J.-H. et al., Anti-GM-CSF Autoantibodies Predict Outcome of Cryptococcal Meningitis in Patients Not Infected With HIV: a Cohort Study, Clinical Microbiology and Infection. (2024) 30, no. 5, 660–665, 10.1016/j.cmi.2024.01.018. 38295989

[21] Arango-Franco C. A. , Rojas J. , Firacative C. et al., Autoantibodies Neutralizing GM-CSF in HIV-Negative Colombian Patients Infected with Cryptococcus gattii and C. neoformans, Journal of Clinical Immunology. (2024) 44, no. 7, 10.1007/s10875-024-01757-y. PMC11249431 39008214

[22] Viola G. M. , Malek A. E. , Rosen L. B. et al., Disseminated Cryptococcosis and Anti-Granulocyte-Macrophage Colony-Stimulating Factor Autoantibodies: an Underappreciated Association, Mycoses. (2021) 64, no. 6, 576–582, 10.1111/myc.13247. 33476401 PMC11913175

[23] Perrineau S. , Guery R. , Monnier D. , Puel A. , and Lanternier F. , Anti-GM-CSF Autoantibodies and Cryptococcus neoformans Var. Grubii CNS Vasculitis, Journal of Clinical Immunology. (2020) 40, no. 5, 767–769, 10.1007/s10875-020-00796-5. 32488475

[24] Trapnell B. C. , Nakata K. , Bonella F. et al., Pulmonary Alveolar Proteinosis, Nature Reviews Disease Primers. (2019) 5, no. 1, 10.1038/s41572-019-0066-3. 30846703

[25] Punatar A. D. , Kusne S. , Blair J. E. , Seville M. T. , and Vikram H. R. , Opportunistic Infections in Patients with Pulmonary Alveolar Proteinosis, Journal of Infection. (2012) 65, no. 2, 173–179, 10.1016/j.jinf.2012.03.020. 22484272

[26] Kavuru M. S. , Malur A. , Marshall I. et al., An Open-Label Trial of Rituximab Therapy in Pulmonary Alveolar Proteinosis, European Respiratory Journal. (2011) 38, no. 6, 1361–1367. 21478218 10.1183/09031936.00197710 PMC3874725

[27] Wu X. K. and Lin Q. , Pulmonary Alveolar Proteinosis Complicated with Nocardiosis: A Case Report and Review of the Literature, World Journal of Clinical Cases. (2021) 9, no. 12, 2874–2883, 10.12998/wjcc.v9.i12.2874. 33969072 PMC8058687

[28] Chiller T. , Farrokhshad K. , Brummer E. , and Stevens D. A. , Effect of Granulocyte Colony-Stimulating Factor and Granulocyte-Macrophage Colony-Stimulating Factor on Polymorphonuclear Neutrophils, Monocytes or Monocyte-Derived Macrophages Combined with Voriconazole Against *Cryptococcus neoformans* , Medical Mycology. (2002) 40, no. 1, 21–26, 10.1080/714031074. 11860010

[29] Browne S. K. , Zaman R. , Sampaio E. P. et al., Anti-CD20 (Rituximab) Therapy for Anti-IFN-γ Autoantibody-Associated Nontuberculous Mycobacterial Infection, Blood. (2012) 119, no. 17, 3933–3939, 10.1182/blood-2011-12-395707. 22403254 PMC3350360

